# Supplementary material for: Structural and configurational properties of nanoconfined monolayer ice from first principles
Source: Sci Rep. 2016 Jan 5;6:18651. doi: 10.1038/srep18651 (PMC4700474; doi:10.1038/srep18651)
Supplement: Supplementary Information [file srep18651-s1.pdf]

Scientific Reports

Supplementary Information for:
Structural and configurational properties of nanoconfined monolayer ice from first principles
Fabiano Corsetti, Paul Matthews, Emilio Artacho

SUPPLEMENTARY NOTE 1

The point dipole and point charge models. Every allowed configuration for the square network (SN) can be idealized as a 2D square grid of point dipoles of magnitude p, with lattice spacing s. Supplementary Fig. 1 shows this for the Aa/Aa configuration. We can separate the molecular dipoles into a three-site point charge model (also shown in the figure), in which there is one free parameter, α, denoting the fractional distance of the protons along the OO bond. The magnitude of the molecular dipole is therefore $p = \sqrt{2}\alpha s q$. $\alpha = 1/2$ results in an arrangement of symmetrized bonds, which is equivalent for all SN configurations. Small deviations from this limit correspond to an alternative point dipole model of bond dipoles (also shown in the figure), with a smaller magnitude $p/\sqrt{2} = \alpha s q$ (where $\alpha \to 1/2 - \alpha$).

We are interested in the difference in electrostatic energy ΔE between two SN configurations. We use the point charge model for this analysis, noting the connection to the dipole models at the two limits:

$$\frac{s}{q^2} \left. \frac{\partial^2 \Delta E^{\mathrm{pc}}}{\partial \alpha^2} \right|_{\alpha=0} = \frac{4s^3}{p^2} \Delta E^{\mathrm{md}} \tag{S1}$$

and

$$\frac{s}{q^2} \left. \frac{\partial^2 \Delta E^{\mathrm{pc}}}{\partial \alpha^2} \right|_{\alpha=1/2} = \frac{4s^3}{p^2} \Delta E^{\mathrm{bd}}, \tag{S2}$$

where E^{pc}, E^{md}, and E^{bd} are the energies from the point charge model, the molecular dipole model, and the bond dipole model, respectively. These relations can be derived by equating the second derivative in α of ΔE^{pc} and ΔE^{md} at the appropriate limit, and using the fact that $\Delta E^{\mathrm{md}} \propto p^2$, and, hence, that $\partial^2 \Delta E^{\mathrm{md}} / \partial \alpha^2 = 2 \Delta E^{\mathrm{md}} / \alpha^2$ (and similarly for ΔE^{bd}).

In order to calculate the energy difference between configurations, we group the point charges as follows:

- the O sublattice as a single group;
- the protons along one row of the SN (each row forming an independent group);
- similarly, the protons along each column of the SN.

The proton grouping reflects the fact that, for allowed configurations, every row and column is independent, with all the protons oriented in the same direction (+ve/-ve) along the line. This means that every water molecule at position (i, j) on the grid has one proton in group Ri and the other in group Cj, as shown in Supplementary Fig. 1. The electrostatic energy for a particular configuration is given by:

$$E^{\mathrm{pc}} = E^{\mathrm{O}} + \sum_i \left[E^{\mathrm{R}i} + E^{\mathrm{C}i} + E^{\mathrm{OR}i} + E^{\mathrm{OC}i} \right] + \frac{1}{2} \sum_{i \neq j} \left[E^{\mathrm{R}i\mathrm{R}j} + E^{\mathrm{C}i\mathrm{C}j} \right] + \sum_{i,j} E^{\mathrm{R}i\mathrm{C}j}, \tag{S3}$$

where E^{X} is the energy within group X, and E^{XY} is the energy between groups X and Y. When considering the difference between two configurations A and B, most of these terms are unchanged and so cancel exactly: E^{O} (the O sublattice is fixed), $E^{\mathrm{R}i}$, $E^{\mathrm{C}i}$, $E^{\mathrm{C}i}$, $E^{\mathrm{OR}i}$ (for a single row or column, a switch in orientation can be seen simply as a reflection across the y- or x-axis, respectively), and $E^{\mathrm{R}i\mathrm{C}j}$ (similarly, switching orientation for the row is a reflection across the column, and vice versa). The only terms remaining, therefore, are:

$$\Delta E^{\mathrm{pc}} = \frac{1}{2} \sum_{i \neq j} \left[\left(E_{\mathrm{A}}^{\mathrm{R}i\mathrm{R}j} - E_{\mathrm{B}}^{\mathrm{R}i\mathrm{R}j} \right) + \left(E_{\mathrm{A}}^{\mathrm{C}i\mathrm{C}j} - E_{\mathrm{B}}^{\mathrm{C}i\mathrm{C}j} \right) \right]. \tag{S4}$$

This gives us an energetic decoupling between rows and columns. The two behave identically, so we proceed only referring to rows. Since every row is defined entirely by its orientation, there will be a cancellation of terms whenever

the relative orientation of Ri with respect to Rj is the same for both A and B (i.e., both in the same direction, or in opposite directions).

The energy difference is therefore given entirely by pairs of rows (i, j) for which the relative orientation differs between A and B. If two rows have the same orientation, the energy per molecule for an infinite lattice is:

$$E^{++}(n) = \frac{q^2}{8\pi\varepsilon_0 s} \sum_{k=-\infty}^{\infty} \frac{1}{\sqrt{k^2 + n^2}}, \tag{S5}$$

where $n = i - j$ (i.e., the number of lattice spacings separating the two rows), and the expression is independent of α. Instead, if they have opposite orientations, it is:

$$E^{+-}(n, \alpha) = \frac{q^2}{8\pi\varepsilon_0 s} \sum_{k=-\infty}^{\infty} \frac{1}{\sqrt{(k + 2\alpha)^2 + n^2}}. \tag{S6}$$

The energy difference $\Delta E^{\mathrm{pc}}(n, \alpha)$ between theses two possibilities is zero at $\alpha = 0$ (the molecular dipole limit), and the function is even and periodic in α with a period of 1/2 (the bond dipole limit). These conditions are sufficient to ensure that energy differences calculated with the two dipole models must be identical. An expression for these can be calculated from the second derivative of the point charge model:

$$\frac{\partial^2 \Delta E^{\mathrm{pc}}}{\partial \alpha^2} = \frac{q^2}{8\pi\varepsilon_0 s} \sum_{k=-\infty}^{\infty} \left[\frac{4}{\left((k + 2\alpha)^2 + n^2\right)^{3/2}} - \frac{12(k + 2\alpha)^2}{\left((k + 2\alpha)^2 + n^2\right)^{5/2}} \right]; \tag{S7}$$

hence, using equations S1 and S2,

$$\Delta E^{\mathrm{md}}(n) = \Delta E^{\mathrm{bd}}(n) = \frac{p^2}{8\pi\varepsilon_0 s^3} \sum_{k=-\infty}^{\infty} \left[\frac{1}{(k^2 + n^2)^{3/2}} - \frac{3k^2}{(k^2 + n^2)^{5/2}} \right]. \tag{S8}$$

This difference is positive, indicating that an opposite orientation of rows is more stable, and exponentially decaying with n (Supplementary Fig. 2(a)). Furthermore, for the point charge model the difference is positive for all α (Supplementary Fig. 2(b)). Neglecting the very small contributions from $n \geq 2$, the total energy difference between A and B depends linearly on the difference between the number of neighbouring rows with the parallel orientations for the two configurations. Therefore, within this approximation, the ratios in energy differences between multiple configurations will be constant for any value of α.

SUPPLEMENTARY FIGURES

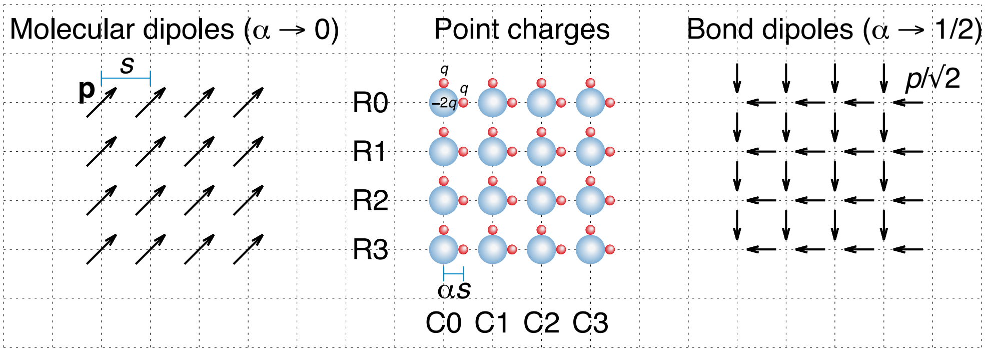

SUPPLEMENTARY FIG. 1. **Point dipole and point charge models of SN monolayer ice.** The Aa/Aa configuration is represented here.

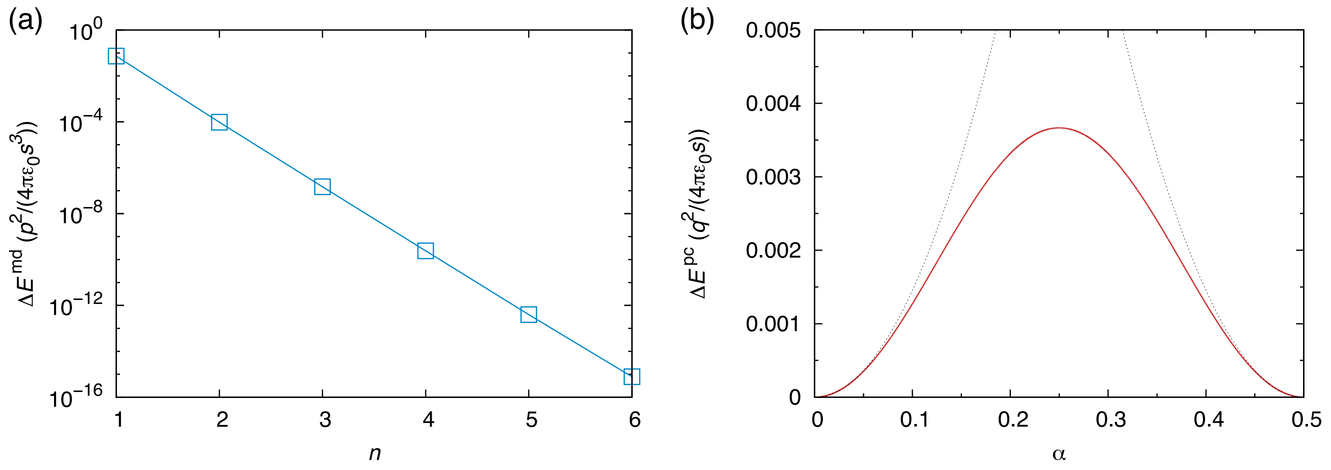

SUPPLEMENTARY FIG. 2. **Energy penalty for pairs of rows with the same proton orientation.** For (a), the value is shown for the dipole model as a function of the number of lattice spacings separating the two rows. For (b), the value is shown for the point charge model for a neighbouring pair of rows ($n = 1$) as a function of the fractional distance of the proton along one lattice spacing; the dotted lines show the energy calculated from the dipole models at the two limits. The corresponding plot for $n = 2$ is not visible on this scale.

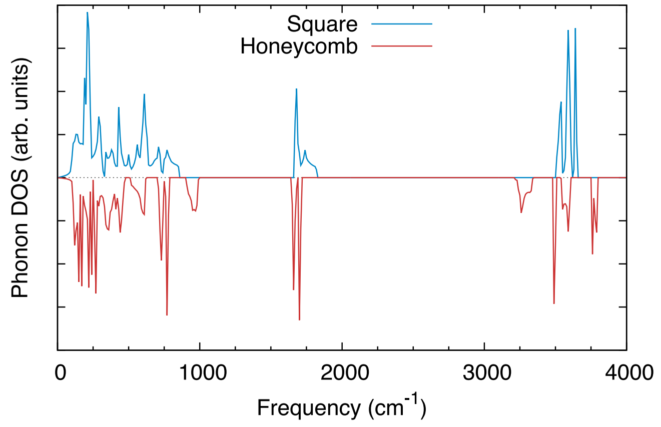

SUPPLEMENTARY FIG. 3. **Phonon density of states for the two networks.** These are calculated for the lowest-enthalpy configuration for each at $d = 5$ Å, $P_l = 0.01$ GPa·Å. A (150×150) grid of q points is used.
